# Automated Flow Cytometry: An Alternative to Urine Culture in a Routine Clinical Microbiology Laboratory?

**DOI:** 10.1155/2017/8532736

**Published:** 2017-09-27

**Authors:** Patricia Mejuto, Mariam Luengo, Julio Díaz-Gigante

**Affiliations:** ^1^Department of Microbiology, Hospital del Oriente de Asturias Francisco Grande Covián, Arriondas, Asturias, Spain; ^2^Department of Biochemistry, Hospital del Oriente de Asturias Francisco Grande Covián, Arriondas, Asturias, Spain

## Abstract

The urine culture is the “gold standard” for the diagnosis of urinary tract infections (UTI) but constitutes a significant workload in the routine clinical laboratory. Due to the high percentage of negative results, there is a need for an efficient screening method, with a high negative predictive value (NPV) that could reduce the number of unnecessary culture tests. With the purpose of improving the efficiency of laboratory work, several methods for screening out the culture-negative samples have been developed, but none of them has shown adequate sensitivity (SE) and high NPV. Many authors show data about the efficacy of flow cytometry in the routine clinical laboratory. The aim of this article is to review and discuss the current literature on the feasibility of urine flow cytometry (UFC) and its utility as an alternative analytical technique in urinalysis.

## 1. Introduction

Urinary tract infections (UTIs) are among the most common infections in both hospitalized patients and outpatients [1–4], and the health cost they cause is considerable. About 50% of women state that they have experienced one infection in their lifetime and 27–48% of them have recurrent infections [5], mainly in senior women living in the community [6]. Although for most patients the disease of the infection is minimal, in certain population groups like children, pregnant women, the elderly, and immunosuppressed patients, it is associated with severe complications [7]. In the USA, it is responsible for more than 7 million medical visits annually, and the most recent medical literature indicates a global prevalence of 0.7% of community-acquired UTI (19.6% in Europe, 12.9% in the USA, and 24% in developing countries) [8]. According to medical literature,* E. coli* more usually causes lower community-acquired UTI [9, 10]. Up to 15% of antibiotics prescribed in the community are due to these infections, with an annual cost of approximately 1.6 billion US dollars [11]. Taking these data into account, there is an enhanced risk of antibiotic resistance [12].

The diagnosis of UTI is based on clinical signs and symptoms of the patient and urinalysis results. Consequently, urine samples are the most numerous specimens received by clinical microbiology laboratories. Culturing of the urine samples is the “gold standard” method to exclude these infections, although it is a significant workload and time-consuming in the microbiology laboratory [13]. Nevertheless, a high percentage of these specimens will yield no growth, reaching even up to 60% of negative results [14, 15]. Also, other different urinalysis techniques are widely used in routine laboratories. The dipstick chemical test is based on strips that had reagent pads for semiquantitative assessment of nitrite (a product of common urinary pathogens), leukocyte esterase, protein, and blood (as a sign of inflammation). This method has a poor NPV, which makes it unsuitable to exclude the presence of urine infection. It is only useful in populations when the results of both nitrite and leukocyte esterase are positive [16]. However, with a negative result, UTI cannot be excluded mainly in children, who normally have low bacterial counts [17]. Another technique is the microscopic examination of urine sediment where cells, particles, and microorganism are counted and reported according to the number observed. Diverse factors can produce interpretation errors, and there are different results among laboratories, due to interindividual variability [18]. Although these screening methods are primarily used in general practice and microbiology laboratories, they are subjective and time-consuming or have a poor sensitivity and/or specificity. For ten years ago, the use of flow cytometry analyzers (FCA) based on detection and quantification both leukocytes and bacteria have been under evaluation. These techniques would reduce the number of samples cultured with a sharp decrease in workload, time, and costs especially in the bigger clinical laboratories [19]. In this way, negative results could be informed considerably earlier, which would imply a reduction of unnecessary empirical antibiotic prescriptions [20].

## 2. Review Methods

Data from 2009 to 2017 were assessed. The search process was conducted using Cochrane, the electronic databases Pubmed/NCBI, and Google Scholar. The terms “urine flow cytometer,” “flow cytometry,” “urinary tract infection,” “screening urinary tract infection” and “flow cytometry analyzers” were used. We also searched reference lists of articles to identify supplementary information. Citations were limited to studies conducted in humans and using the UF1000i (Sysmex) analyzer for UTI screening and the urine culture as the reference standard. Conference abstracts were excluded due to lack of information provided. Our initial literature search yielded 605 unique citations. After a critical evaluation, based on study design, a total of 17 articles were selected, for review (Table 1).

## 3. Diagnosis of Urinary Tract Infection

The detection of the uropathogen by urine culture is the “gold standard” for the diagnosis of UTI. In this way, the level of the bacteriuria can be estimated and the antimicrobial susceptibility determined. However, the minimum number of bacteria demonstrating an infection of the urinary tract has not been specified in the scientific literature or fixed by microbiological laboratories [37, 38]. In 1960, Kass developed the concept of significant bacteriuria as more than 10^5^ CFU/mL when pyelonephritis happens during pregnancy [39]. This assessment introduced quantitative microbiology into the diagnosis of infectious diseases and is therefore still of general importance. But, there is no standardized bacterial count that indicates significant bacteriuria, applicable in all types of UTIs.

The European urinalysis guidelines recommend the following limits of uropathogens counts, for a diagnosis of UTI [36, 40]: a threshold of >10^5^ Colony Forming Unit (CFU) per ml in MSU (midstream urine) in women or >10^4^ CFU/ml in MSU in men or straight urine catheter in complicated UTI in women; more than 10^4^ CFU/ml in a MSU in uncomplicated pyelonephritis in women or >10^3^ CFU/ml in MSU in uncomplicated cystitis in women. In a sample of urine obtained by bladder puncture, any count is significant. According to the Infectious Diseases Society of America (IDSA) guideline, for uncomplicated UTI, counts of more than 10^4^ CFU/ml can be considered as the cut-off for positive culture [41, 42].

## 4. Urinary Flow Cytometer Principle

Flow cytometer analyzers are systems for the rapid, optical analysis of individual cells that allow simultaneous analysis of chemical and/or physical characteristics of single urinary particles [43]. Measurement is made by an array of detectors as the cells flow in a fluid stream through a laser beam. The urinary flow cytometer (UFC) can identify red blood cells (RBC), white blood cells (WBC), squamous epithelial cells, small round cells (renal tubular cells and transitional epithelial cells), hyaline casts, bacteria, yeast-like cells, spermatozoa, and crystals. The urine sample is automatically mixed, aspired, and stained using a specific fluorescent polymethine dye in two different analytical chambers: one for bacteria and another one for other urine forming elements. Once stained, the fluorescent particles are inserted into the cytometer pool and exposed to the semiconductor laser beam (*ƛ* 635 nm). For each particle the scattered light is detected at two positions: forward and side scatters and fluorescence intensities are produced [44, 45]. These signals are converted to optoelectronic signals so that components can be identified, counted, classified, and analyzed. Bacteria are counted via an individual bacterial examination channel, so any interference with RBCs is prevented. All the results are presented in histograms and scattergrams by the software. Each particle type is color coded for the ease of reading. The bacteria and WBC counts are recorded in a database for subsequent analysis to determine the optimal screening method.

In recent years, the analytical quality of these analyzers has been improved because bacteria are counted in an independent examination channel, and interference with red blood cells is prevented. Also, the latest models have a more powerful laser that provides a high sensitivity analytic as well as a flag which informs on the gram characteristics of bacteria detected.

## 5. Studies

Several studies reported different sensitivities and specificities about the application of flow cytometry in the diagnosis of suspected UTI. The results obtained depend on the definition used for gold standard positive and negative urines cultures, and these definitions vary among laboratories. For example, most often >10^5^ CFU/ml [22, 25, 27, 29, 46] has been considered a threshold for positive urine cultures in the clinical, microbiological practice. Some authors used pathogenicity cut-offs of 10^3^ CFU/ml or less [28, 47, 48] while others consider high counts of 10^5^ CFU/ml that were still measured negative [49]. Taking into account these microbial counts limits, authors establish different cut-off values of bacteria and leukocytes per *μ*l, below which the reported result of the urinalysis will be negative. We have observed that when increasing the definition of a negative urine culture, the cut-points that the authors use for screening are higher. In the same way, these combinations of cutoff values might improve the combination of sensitivity and specificity of detecting UTI by the analyzer. For example, Broeren et al. [14] reported a SE of 96%, a SP 78%, and a NPV of 99.7%, at a cutoff value of 230 bacteria/*μ*l, when a definition of negative urine culture of <10^5^ CFU/ml was used. When these authors choose a less stringent gold standard definition (<10^4^ CFU/ml), the cutoff decreases until 39 bacteria/*μ*l, with a consequent decrease in SE (82%) and an increase of SP (83%). Other studies, with the same definition of negative urine culture (<10^5^ CFU/ml), show different data. Manoni et al. [27] choose cut-off values for bacteria and leukocytes of 125/*μ*l and 40/*μ*l, respectively, to obtain a sensibility of 97% with a specificity of 94%. Gutiérrez-Fernández et al. [29] fix different counts limits (690 bacteria/*μ*l and 38 leukocytes/*μ*l), mainly due to differences in the population analyzed.

On the other hand, in two studies, we observed strikingly low cut-off points. Kadkhoda et al. [30] considered >20 bacteria/*μ*l, using a definition of culture positive of >10^5^ CFU/ml for uropathogens. Krongvoraluk et al. [50] showed the lowest cutoffs of the revised literature (bacteria >14.2/*μ*l and leukocyte >6.7/*μ*l), perhaps for the use of the filter paper method instead of the calibrated loop urine quantification technique.

Diverse papers have shown that the use of FCA reduces the number of processed negative urinary specimens. Authors demonstrate variably reduction in bacterial culture ranging from 28% to 60% [14, 22, 24–26, 29–33, 50]

We observed a wide variation in cut-off points applied, as well as in the SE and SP of the results obtained in the literature. Several authors did not recommend to use a standard, unique cutoff value of both bacteria and leukocytes counts. These will be chosen depending on the characteristics of the analyzed population, which is usually heterogeneous. Furthermore, published data recommended adjusting the cutoff values according to gender and age of the patients to increase detection reliability [15, 23, 31, 51].

If confirmed in studies with larges series, the UFC screening method would be more efficient when both bacterial and leukocyte counts were detected. Various authors demonstrate that this implies an increase in the sensitivity of the technique [24, 29, 33, 35, 49]. In contrast to these findings, other studies do show no benefit when combining the use of both counts for UTI screening [14, 26, 30].

Also, some authors suggest that, with the general definition of <10^5^ CFU/ml for a negative urine culture, a significant amount of microorganisms would not be considered. But, down CFU counts (≤10^3^ CFU/ml) in some specific groups of patients (urological patients, children, pregnant women, and catheterized patients or when “fastidious” microorganisms are involved) are relevant. For this reason, some authors suggest that these samples have to be evaluated individually and also cultivated [14, 27, 30].

On the other hand, diverse studies with similar patient population analyzed, and the same gold standard definition for negative culture, show differences in the number of false negative results [14, 27, 33]. Diverse aspects can explain these discrepancies. Defects in the cytometer detection of Gram-positive bacteria, due to phenomena of aggregation, have been described by several authors [47, 49, 52]. There are other factors than can lead to high count false negatives: antibiotic treatment before urine analysis, for nonviable bacteria detection [14] as well as different culture procedures, transportation times or the use of boric acid as preservative [19].

We checked that the majority of subjects analyzed were outpatients [14, 22, 26, 29, 31, 35], whereas other authors evaluated mainly samples of hospitalized patients [28, 53]. Besides, most studies did not establish the discrepancies between hospitalized patients and outpatients. But, it is important to distinguish them because there are substantial differences, in connection with the presence of catheters or the use of antimicrobials [19].

Concerning to microorganisms causing UTI, Gram-negative bacteria were reported more frequently, being* E. coli* the most prevalent microorganism isolated in samples with significant bacteriuria. In different series analyzed, it represents more than 50% of isolates [27, 30, 33, 52]. Curiously, Monsen and Rydén [21] confirmed that different microorganisms were associated with distinct parameters counts.* Proteus mirabilis*,* P. aeruginosa, S. aureus*, and group C streptococci showed high leukocyte counts.* Klebsiella *sp. had smaller leukocyte counts whereas the highest bacterial, leukocyte, and erythrocyte counts were found for* E. coli. *One study conducted in Spain [22] demonstrated that cultures with* E. coli* and* Klebsiella *spp. had less epithelial cells counts that contaminated urine samples. And specimens with* Enterococcus *spp. were associated with lower WBC counts than negative urines.

From an economical point of view, some studies have examined the possible savings that would be obtained with the use of this automated system in routine clinical microbiology laboratories. Marschal et al. [28] demonstrated money savings of 5965 euros every 12000 urine samples per year. And in one study conducted in Turkey, Yasuma et al. [54] showed savings of 239–306 US dollars per 100 samples. Ilki et al. confirmed that the method is cost efficient since it represents savings of at least 6798 euros [55].

Finally, in most of the eligible studies it was unknown if patient's clinical manifestations were representatives of UTI. On the other hand, criteria of inclusion and exclusion were not determined as well as information about preanalytical phase. Also, only a few studies reported the percentage of contaminated samples and its influence in UTI prevalence.

## 6. Conclusions

It is not possible to provide effective patient management if the results of urinary investigation are not interpreted considering patient's clinical characteristics. It is important to remember that the number of bacteria deemed relevant for the diagnosis of UTI depends on various factors. The type of specimen (mid-stream urine, bag urine, catheter urine, urostomy urine, and suprapubic aspiration urine), clinical situation of the patient (abnormalities of the genitourinary tract, presence of clinical manifestations, gender, age, pregnancy, and antibiotics previously taken), or variety of bacteria (category and isolated species) should be considered [19]. With a unique gold-standard definition for a negative culture of <10^5^ UFC/ml, the presence of high bacteria amount is considered not significant. That is questionable, especially for particular patients as well as children, pregnant women, and older adults, for whom low counts are relevant.

Classical microbiology techniques are quite slow in comparison to new automated methods, based on advanced technologies for UTI screening, as they require the isolation of the organism before its identification and subsequently antibiotic susceptibility determination. With the implementation of FCA in clinical laboratories, the results are practically available in real time, within a few minutes of the sample admission and can be promptly informed in case of negative results.

It is vital to bear in mind that sensitivity is the most important requirement of a good screening process. In a screening test all positive specimens are cultured and, consequently, false-positive results will not be informed to the clinicians [27]. For FC to be a useful rapid test to rule out negative urine samples, the attention should be paid to obtaining the optimal results in comparison with the urine culture. This would require the highest sensibility and therefore a high negative predictive value, to minimize the number of false negative results.

Different limits for both leukocytes and bacteria detection were established in available reports. Each microbiology laboratory should adjust its bacteria and leukocytes cutoffs, depending on their UTI prevalence and the pathology of the patients analyzed as well as their origins, gender, and age.

In patients with UTI, the TAT (turnaround time) for a test result is approximately 48 h. For a patient with overt clinical manifestations, it is too long, because the introduction of antibiotic therapy should be early. Unfortunately, the most active antibiotics against Gram-positive bacteria are not useful in Gram-negative germs, which are the most frequent bacteria involved in urinary tract infections. Differentiating the type of bacteria involved in these infections would help to improve the efficacy of empirical therapy [49, 56, 57]. If information about the Gram properties of the suspected bacteria was available quickly, clinicians could select the most effective antibiotic for UTI infection.

Most revised articles have agreed that FC is a useful method for screening urinary samples if the optimal cut-off value is established for each group of patients. The adoption of correct criteria is crucial to improving the performance of the screening process. This will lead to significant reduction in TAT of negative cultures in bacterial culture and unnecessary empirical antibiotic prescription. However, in subjects at risk of UTI complications, its use is controversial, being the culture necessary.

In the future, perhaps the possibility of combining these systems with a rapid identification technique like matrix-assisted laser desorption ionization time-of-flight (MALDI-TOF) mass spectrometry (MS) can permit recognizing bacterial species directly in urine positive samples [58–60] and choosing the best antibiotics depending on the results provided by the analyzer. Further studies could be of interest to investigate the impact of these automatic techniques in both prescription of antibiotics and its relationship with patient care.

At present, there is a need to improve the quality and optimize the procedures performed in laboratories. Before deciding to automate a manual technique, a rigorous study should be conducted. In this way, microbiologists could offer the best diagnostic option to patients and physicians.

## Figures and Tables

**Table 1:** Summary of eligible studies.

Reference (country)	Patients and samples	Reference Counts	Cutoff Values	SE/SP/NPV/ER%
Monsen and Rydén 2015 (Turkey) [21]	4458 urine samples from adults	>10^4^ CFU/mL	50 BAC/*µ*L 30 WBC/*µ*L	1/49.7/1/44.8

García-Coca et al., 2017 (Spain) [22]	17483 urine samples from 85% women and 14.7% (median age 47 years). 97.4% outpatients and 2.6 hospitalized subjects	>10^5^ CFU/mL	>200 BAC/*µ*L >40 WBC/*µ*L	AUC (BAC) count: 0.79 AUC (WBC) count: 0.60 ER: 48

Kulkarni and Nigrin 2013 (Netherlands) [23]	7322 urinary samples from 60% inpatients and 40% outpatients (average age 66 years)	>10^5^ CFU/mL	>200 BAC/*µ*L	0.93/0.63/0.96/49

Íñigo et al. (2016) [24] (Spain)	1934 urine samples: MSU (89.14%), catheterized urine (9.98%), pediatric urine bags (0.72%), suprapubic aspiration (0.16%), belonging to 56% ♀ and 44% ♂ (51.19% hospitalized patients and 48.81% outpatients)	>10^2^ CFU/mL	460 BAC/*µ*L 40 WBC/*µ*L	0.98/0.76/0.99/57

Gessoni et al., 2015 (Italy) [25]	2335 MSU from adult patients (41% inpatients and 59% outpatients), between 15 to 91 years old (♀: 34%, ♂: 66%)	≥10^5^ CFU/mL	175 BAC/*µ*L	0.95/0.80/—/50

Martín-Gutiérrez et al., 2015 (Spain) [26]	346 MSU collected from elderly outpatients with a median age of 76 years (♀: 43%, ♂: 56%)	≥10^5^ CFU/mL for women ≥10^4^ CFU/mL for men	200 BAC/*µ*L	0.99/0.91/0.99/60

Manoni et al., 2009 (Italy) [27]	372 MSU specimens from 167 ♂ and 205 ♀ (57.37% outpatients and 42.63% inpatients)	>10^5^ CFU/mL	14.2 BAC/*µ*L 6.7 WBC/*µ*L 14.2 BAC/*µ*L	0.8/0.58/0.86/—/— 0.95/0.44/0.95/30.6

Marschal et al., 2012 (Germany) [28]	572 urine samples (31.8% catheterized, 65.5% MSU and 2.7% not specified), from 513 patients (27.9% outpatients, 72.1% inpatients) ♀: 48.9% and ♂: 51.1% (mean age of 54.2 years)	>10^2^ CFU/mL	>30 BAC/*µ*L >20 WBC/*µ*L	0.80/0.78/—/—

Gutiérrez-Fernández et al., 2012 (Spain) [29]	1225 urine samples (urine catheter, MSU or pediatric bags) from 158 inpatients (27 catheterized adults, 33 children, 98 inmunocompromised adults) and 1067 outpatients (464 adults and 603 pregnant women).	>10^5^ CFU/mL	690 BAC/*µ*L 38 WBC/*µ*L	0.92/0.65/0.97/—

Broeren et al. (2011) (Netherlands) [14]	1577 urine samples collected from 681 outpatients (196 ♂) and 896 (403 ♂) from hospitalized patients	<10^5^ CFU/mL <10^4^ CFU/mL	230 BAC/*µ*L 39 BAC/*µ*L	0.96/0.78/0.99/52 0.82/0.83/0.95/28

Kadkhoda et al. 2011 (Canada) [30]	2496 (75%) MSU and 25% catheterized urine (patients origin not informed)	≥10^4^ CFU/mL of up two uropathogens ≥10^5^ CFU/mL Non uropathogens	20 BAC/*µ*L	0.93/0.69/—/35

Pieretti et al. 2010 (Italy) [31]	703 MSU from 18.2% hospitalized patients and 81.8% outpatients (70.6% females and 29.4% males)	>10^4^ CFU/mL	65 BAC/*µ*L 100 WCB/*µ*L	0.98/0.62/0.98/43

van der Zwet et al. 2010 (Dutch) [32]	358 urine samples (patients origin not informed)	>10^4^ CFU/mL	50 BAC/*µ*L 20 WBC/*µ*L	1/0.8/0.59/42

De Rosa et al. 2010 (Italy) [33]	1349 urine samples (patients origin not informed)	>10^4^ CFU/mL for adult >10^6^ CFU/mL for children.	170 BAC/*µ*L 150 WBC/*µ*L	0.98/0.76/0.99/57

Shang et al. 2013 (China) [34]	313 urine samples, belonging inpatients and outpatients	>10^5^ CFU/mL for gram (−) >10^4^ CFU/mL for gram (+)	100 BAC/*µ*L 56 WBC/*µ*L	0.86/0.95/0.94/50

Hu et al. 2010 [35] (China)	308 urine samples from outpatients	>10^5^ CFU/mL for gram (−) >10^4^ CFU/mL for gram (+)	160 BAC/*µ*L	0.81/0.83/0.89/55

Kouri 2000 (Italy) [36]	1463 MSU samples from adults	>10^5^ CFU/mL	>125 BAC/*µ*L >40 WBC/*µ*L	0.99/0.77/0.98/—

MSU: midstream urine sample; WBC: white blood cells; BAC: bacteria, NPV: negative predictive value; CFU: colony forming units; ER: elimination rate; AUC: area under the ROC curve.
